# Healthcare utilization, quality of life, and work productivity associated with primary hyperoxaluria: a cross-sectional web-based US survey

**DOI:** 10.1007/s00240-023-01436-4

**Published:** 2023-04-17

**Authors:** David S. Goldfarb, Frank Modersitzki, John Karafilidis, Josephine Li-McLeod

**Affiliations:** 1 New York University Grossman School of Medicine, New York, NY 10016 USA; 2 Dicerna Pharmaceuticals, Inc., a Novo Nordisk Company, Lexington, MA USA; 3 Stratevi, Boston, MA USA

**Keywords:** Primary hyperoxaluria, Quality of life, Work productivity, Activity impairment, Patient involvement, School attendance

## Abstract

Primary hyperoxaluria (PH) is a family of ultra-rare, autosomal recessive, metabolic disorders associated with frequent kidney stones, chronic kidney disease and kidney failure, and serious complications due to systemic oxalosis, resulting in significant morbidity. We investigated the burden of PH among affected patients and caregivers. This cross-sectional, web-based survey was used to quantify the burden of PH, in terms of healthcare resource utilization, health-related quality of life, and work productivity and activity impairment among adults (≥ 18 years) with PH and caregivers of children (≤ 17 years) with PH in the US. Among the 20 respondents, there were 7 adults with PH and 13 caregivers of children with PH. Adherence to hyperhydration was noted as the most, or one of the most, difficult aspects of PH by 56% of respondents. Most patients (95%) had experienced painful kidney stone events, one-third had visited the emergency room, and 29% were hospitalized for complications due to PH. Of the 24% of patients on dialysis, all found the procedure burdensome. Adult patients’ quality of life was negatively affected across several domains. Most respondents (81%) reported that PH had a negative effect on their finances. Employed adult patients and caregivers, and children with PH, had moderate impairment in work productivity, school attendance, and activity. Anxiety about future PH-related sequelae was moderate to high. These findings highlight the need for improvements in PH medical management. A plain language summary is available in the supplementary information.

**Supplementary Information:**

The online version contains supplementary material available at 10.1007/s00240-023-01436-4.

## Introduction

Primary hyperoxaluria (PH) is a family of three ultra-rare, autosomal recessive, metabolic disorders leading to markedly elevated levels of endogenous oxalate in plasma and urine, which is associated with frequent kidney stones, chronic kidney disease and kidney failure, and serious complications due to systemic oxalosis [1–9]. The distinct genetic disorders (PH1, PH2, PH3) together have an estimated prevalence of 1 per 38,630 in the United States (carrier frequency, approximately 1:58) [4]. PH1 accounts for approximately 80% of genetically diagnosed cases [4].

Traditionally, treatment of patients with PH, particularly PH1, involves conservative medical management followed by dialysis if end-stage kidney disease occurs, and ultimately, liver and/or kidney transplant [10]. Supportive medical interventions attempt to mitigate stone formation and slow renal function decline by decreasing urine oxalate concentration and calcium oxalate crystallization [11–13]. This involves aggressive fluid intake, and oral administration of alkali citrate compounds (such as potassium citrate and sodium citrate) and neutral orthophosphate [10–13]. In addition, vitamin B_6_, a glyoxylate aminotransferase AGT cofactor, is capable of reducing urine oxalate excretion in a subgroup of patients with specific mutations in *AGXT* causing PH1 [11].

Self-evidently, the burden of PH exerts an enormous toll on affected patients and their caregivers. PH-related morbidities such as kidney stones cause disabling and acute pain while cumbersome hyperhydration regimens interfere with daily life [2, 12, 14, 15]. Fear of organ transplantation among patients and their caregivers is associated with severe physical, emotional, and financial hardship [14]. However, the extent to which PH-related sequelae and their management can affect daily life is not well established.

Currently available information on the burden of PH for both patients and caregivers largely focuses on disease manifestations and healthcare resource utilization (HRU) in PH1 [1, 14, 16], leaving knowledge gaps regarding the effect of PH on health-related quality of life (HRQOL) and work productivity and activity impairment (WPAI).

The objective of this cross-sectional, web-based survey was to quantify HRU, HRQOL, and work productivity among PH patients and caregivers residing in the United States. The study also assessed the challenges associated with current diagnosis and treatment for PH to better understand the patient and family journey.

## Methods

### Survey design and oversight

The survey was organized jointly by Dicerna Pharmaceuticals, Inc., a Novo Nordisk Company (Lexington, MA), and Stratevi (Boston, MA) after New England Institutional Review Board (now WCG IRB) approval (tracking number, 20201538). The survey was conducted in accordance with data privacy regulations and reported in the spirit of best practice guidelines [17–19]. Approval for entry was voluntary. Informed consent was obtained from each participant, all of whom received US$100 for completion of the quantitative and qualitative surveys. All responses were anonymized to protect patient confidentiality. In addition, the survey complied with national data collection regulations.

### Recruitment and data source

**Fig. 1:**
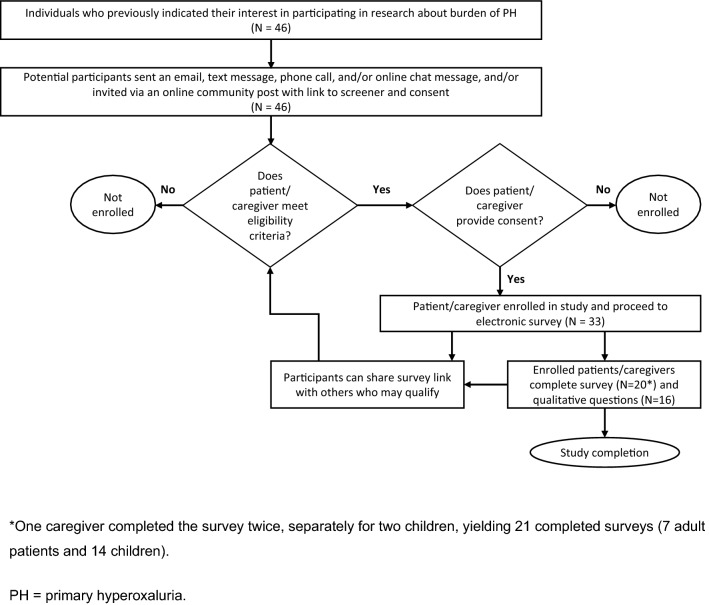
Recruitment of patients and caregivers participating in online survey

Individuals who had expressed an interest in participating in a previous research study about the burden of PH through the Oxalosis and Hyperoxaluria Foundation, a patient support and advocacy organization, were notified about the opportunity to participate in this present study (Fig. 1). Those who accepted the invitation were directed to the landing page of the online survey, detailing aims and assurances around confidentiality and informed consent. Individuals were then asked to complete screening questions to determine their eligibility to participate. Given the rarity of PH, participants were entitled to share the survey link with other potential candidates. The survey was issued with the intention of attaining 30 completed questionnaires.

### Survey participants

The cohort included adults (≥ 18 years) with self-reported PH and self-reported caregivers of children (≤ 17 years) with PH in the United States. To be eligible, the individual with PH had to have been treated for PH or had to have been seen by a doctor for PH in the past 12 months. Key exclusion criteria included a prior liver or kidney transplant for PH, lack of a cell phone that could send and receive text messages or access to a computer, and current or planned enrollment in a clinical trial for PH prior to administration of the survey.

#### Study measures

All participants completing the questionnaire were asked about their or their child’s demographics, clinical characteristics, medical care, HRU, HRQOL, and WPAI. Participants also completed a qualitative voice response survey to gain insights into challenges with the PH diagnosis process, effect of kidney stone events on daily life, current PH treatment regimen and challenges, effect of PH on daily life, and health insurance challenges related to managing PH (Supplemental Figure). The qualitative voice response survey enabled some rationalization of the quantitative data.

#### Healthcare resource utilization (HRU)

HRU was assessed as the number of visits to a range of healthcare providers, emergency room (ER) visits, and hospitalizations in the past 6 months. Estimates of HRU from patient-reported data in several populations bear resemblance to data emanating from retrospective administrative claims databases and physician records [20–24].

#### Health-related quality of life (HRQOL)

HRQOL was assessed by administering the Kidney Disease Quality of Life (KDQOL-36™) [25–27] and Wisconsin Stone Quality of Life (WISQOL) [28, 29] questionnaires, both of which are kidney disease–specific, multidimensional, and psychometrically validated.

The KDQOL-36 is the most widely used survey in patients on dialysis but is also suitable for patients with less severe chronic kidney disease [25–27, 30]. It comprises five subscales as follows: Physical Component Summary (PCS), Mental Component Summary (MCS), Burden of Kidney Disease (BKD), Symptoms and Problems of Kidney Disease (SPKD), and Effects of Kidney Disease (EKD). The PCS and MCS represent the generic core of the KDQOL-36™ (and are identical to the 12-Item Short Form Survey [SF-12]), which is supplemented by the three kidney disease-specific scales [30]. The KDQOL-36 has strong coverage of concepts important for understanding the experience of patients living with chronic kidney disease [31], as it informs on the signs and symptoms of kidney disease as well as the life effects associated with kidney disease [25, 32]. The KDQOL-36 score ranges from 0 to 100, where a higher score corresponds to better self-reported HRQOL.

The 28-item WISQOL questionnaire is designed for patients who form kidney stones, and it can discriminate among patients with different stone statuses and symptoms [28, 29]. Morbidity is measured in four domains specific to nephrolithiasis patients: social effect, emotional effect, disease effect, and effect on vitality [29]. Each item in the WISQOL questionnaire is scored on a Likert-scale ranging from 1 to 5, yielding a maximum score of 140. A higher score on the WISQOL questionnaire indicates a higher self-reported HRQOL.

#### Work productivity and activity impairment (WPAI)

The validated WPAI tool captured the percentage impairment in work and non-work-related activities of daily living during the previous week [33]. The questionnaire generates four subscales as percentages for absenteeism, presenteeism, overall work impairment, and activity impairment, for which in this study, a score of 0 signifies no impairment or productivity loss due to PH and a score of 100% signifies complete impairment or productivity loss due to PH. Data on absenteeism, presenteeism, and overall work impairment were restricted to adult patients and employed caregivers in paid employment, but all respondents provided data on activity impairment.

#### Statistical analyses

The completion rate was the number of individuals submitting the last page of the questionnaire divided by the number of people completing the screener.

Descriptive statistics summarized all variables. Findings from closed-ended questions were presented as incidences, percentages, means, or medians. The number of respondents to whom each question applied was the denominator. Open-ended responses were treated as qualitative data and, when possible, coded into categories.

Responses from the quantitative survey were stratified according to PH type (PH1, PH2, PH3, and unknown), age group (0–10, 11–17, 18–40, and 41–64 years), and PH severity proxy (≤ 2 and > 2 kidney stone events in the past 5 years).

All analyses were conducted using R 3.6.1.

## Results

### Response

Thirty-three individuals completed the screener (Fig. 1). Thirteen of these individuals did not meet eligibility criteria. All 20 eligible individuals completed the survey, including 1 caregiver who completed 2 separate surveys on behalf of their 2 affected children. Thus, among the screened individuals, the questionnaire completion rate was 64%. Only 1 respondent completed the WISQOL questionnaire, and therefore these results are not included in this article. Among the 21 patients included in the study, there were 16 responses for the qualitative voice response survey.

### Respondent characteristics

Details regarding the 21 patients with PH were reported by 7 adults with PH and 13 caregivers of 14 children with PH (Table 1). Most patients with PH were female (71%). All PH types were represented, including 13 adults and children with PH1 (62%), and 3 children each with PH2 (14%) and PH3 (14%) (PH type was unknown for 2 respondents).

**Table 1:** Characteristics of patients with PH and their caregivers

Patients with PH	Status (N = 21)
Female, n (%)	15 (71)
*Age, years, n (%)*	
0–17	14 (67)
18–46	7 (33)
*PH type, n (%)*	
Type 1	13 (62)
Type 2	3 (14)
Type 3	3 (14)
Unknown^a^	2 (10)
Employed among adult patients (n = 7), n (%)	4 (57)
*Age at diagnosis, years (n = 19)*	
Mean (SD)	10.1 (16.3)
Median (min–max)	5.0 (0.2–62.0)
*Health insurance, n (%)*	
Private insurance	12 (57)
Medicaid/CHIP	4 (19)
Medicare	1 (5)
VA or DOD or TRICARE	1 (5)
No health insurance	2 (10)
Prefer not to answer/I do not know	1 (5)

Caregivers	Status (N = 13)^b^
Female, n (%)	12 (92)
Age, years, median (min–max)	38 (27–50)
*Main caregiver, n (%)*	
Yes	6 (46)
Share caregiving equally with another caregiver	7 (54)
Employed, n (%)	9 (69)

*CHIP* Children's Health Insurance Program, *DOD* Department of Defense, *max* maximum, *min* minimum, *PH* primary hyperoxaluria, *VA* veterans affairs

^a^PH type not known at the time of survey administration

^b^One caregiver completed two separate surveys on behalf of their two affected children

Overall, the median (range) age of patients at diagnosis was 5.0 years (0.2–62.0), breaking down to 5.0 years (0.2–37.0) in patients with PH1, 1.3 years (1.0–4.0) in patients with PH2, and 1.8 years (1.2–5.0) in patients with PH3. Patients who had > 2 kidney stone events in the past 5 years (i.e., more severe) were diagnosed at a median age that was 2 years later than those who had ≤ 2 kidney stones in the past 5 years (median, 5 vs. 3 years). In the qualitative voice response survey, 6 of 16 respondents (38%) reported experiencing a misdiagnosis. Twelve of 16 (75%) respondents reported experiencing a kidney stone event as part of their diagnosis journey.

### Medical care

**Fig. 2:**
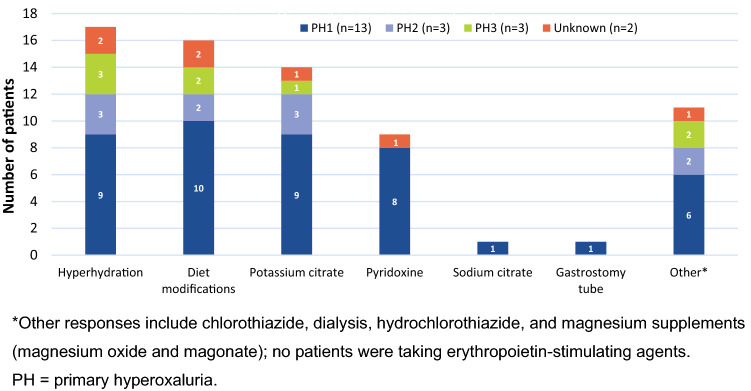
Current management of PH (N = 21)

Figure 2 summarizes the current medical management of the 21 patients with PH, including diet modifications, hyperhydration, and pyridoxine use (PH1 patients only). The amount of water consumed was highest among the 5 patients 11–17 years old (median [range] 115 oz per day [85–136]), with patients 0–10 (n = 9), 18–40 (n = 3), and 41–64 (n = 4) years old consuming a median of 40 oz (range, 20–100), 100 oz (range, 75–106), and 80 oz (range, 24–100) of water per day, respectively. There were no major differences in the amount of water consumed daily by PH severity because both groups consumed a median of 80 oz daily. The median amount of water consumed per day was greater in patients with PH1 (80 oz) and PH2 (85 oz) than in patients with PH3 (40 oz).

In the qualitative voice response survey, 9 of 16 respondents (56%) noted that hyperhydration was the most (or one of the most) burdensome aspects of PH treatment. For all patients < 10 years old (n = 7), hyperhydration was either difficult (n = 5, 71%) or very difficult (n = 2, 29%). The most cited reasons why adherence to hyperhydration is difficult are not wanting to drink or not thirsty, difficulty in remembering to drink, and difficulty drinking large amounts of water.

### Healthcare resource utilization (HRU)

**Fig. 3:**
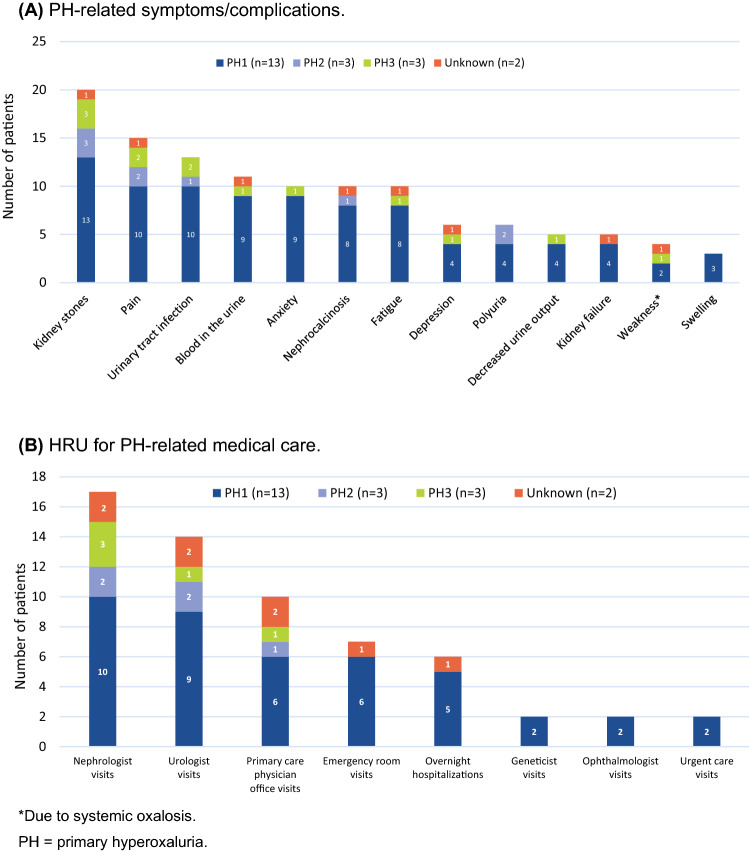
**A** Symptoms and complications ever experienced and **B** healthcare resource utilization (HRU) by patients with PH (N = 21)

PH-related symptoms and complications, and HRU associated with the PH burden, are illustrated in Fig. 3. Almost all patients (n = 20, 95%) experienced a kidney stone event at least once in their lives, with 35% experiencing kidney stone events more than once a year. Most patients (80%) had moderate or severe pain from their last kidney stone event. The more severe group typically had more symptoms/complications than the less severe group. Over the last 5 years, patients experienced a median of two kidney stones: patients with greater PH severity experienced more stone events than those with lower PH severity (median, 8 vs. 1.5 events), and older adults (41–64 years) experienced more stone events than younger patients (11–17 years) (median, 16 vs. 4). In addition, over the last 5 years, approximately half of the kidney stone events experienced by patients required urological stone removal procedures (e.g., lithotripsy, percutaneous nephrolithotomy, and ureteroscopy). The duration of symptoms of the last kidney stone event experienced was 1 week; however, for some patients, symptoms lasted longer (range, 0–20 weeks).

One third of patients (n = 7) visited the ER (most commonly for pain, n = 3), and more than one quarter were hospitalized (n = 6, 29%) (Fig. 3B). Approximately one quarter (24%) of the patient sample (4 children and 1 adult) were either on hemodialysis (n = 3) or peritoneal dialysis (n = 2), which required a median of 24 h per week (range 18–36) spent on treatment. All 4 children on dialysis had PH1. In the qualitative voice response survey, all 4 caregivers whose children were receiving dialysis reported that it was the most or one of the most burdensome aspects of PH treatment.

**Fig. 4:**
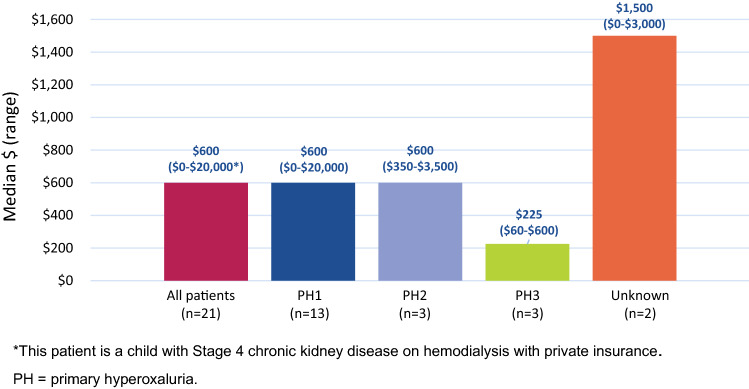
Out-of-pocket expenses for PH health services or medications over the past 6 months

The median out-of-pocket expenses in the past 6 months was $600 and increased to $2000 for children 11–17 years old (Fig. 4). Most respondents (13 of 16; 81%) reported that PH affected their finances, including difficulty paying for healthcare out-of-pocket expenses (primarily premiums and deductibles). Twelve of 16 respondents (75%) reported facing challenges with insurance coverage for PH, including coverage limitations (doctors, specialists, medications, tests), inability to get insurance, and paying for more out-of-pocket expenses for better coverage.

### Health-related quality of life (HRQOL)

**Fig. 5:**
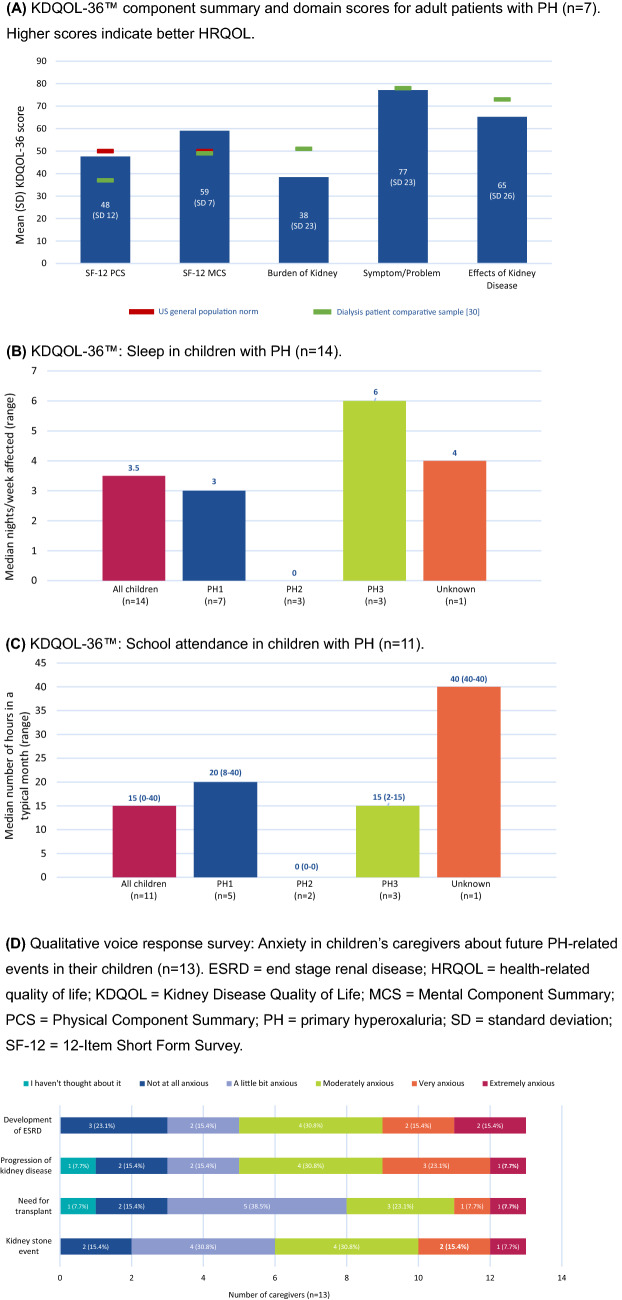
Health-related quality of life summary

On the KDQOL-36™ questionnaire, adult patients with PH (n = 6) had mean PCS and MCS scores of 48 and 59, respectively (Fig. 5A) [30]. Mean KDQOL-36™ scores for burden of kidney disease, symptom/problem, and effects of kidney disease were 38, 77, and 65, respectively.

Caregivers reported that PH negatively affected both their sleep (median, 3.0 nights/week; range, 0–7) and their children’s sleep (median, 3.5 nights/week; range, 0–7) (Fig. 5B). Older children (11–17 years) had more negatively affected sleep than younger children (0–10 years) (median of 4 vs. 0 nights). Children who had > 2 kidney stone events in the past 5 years (i.e., more severe) had more negatively affected sleep than those with ≤ 2 kidney stones (median of 4 vs. 0 nights).

Of the children who attended school (n = 14), a median of 15 h (range, 0–40 h) of class was missed in a typical month due to their PH (Fig. 5C). A median of 20 h (range, 8–40 h) and 15 h (range, 2–15 h) of class was missed in a typical month due to PH1 and PH3, respectively. Children who had > 2 kidney stone events in the past 5 years (i.e., more severe) missed more school in a typical month compared to those with ≤ 2 kidney stones (median of 15.5 vs. 2 h). The prevalence of accommodations provided by school for children with PH was recorded: 82% were allowed to miss school for doctor's appointments or other reasons related to PH, 82% had rest and bathroom privileges, and 73% were allowed to eat and drink during class.

In the qualitative voice response survey, 7 of 16 respondents (44%) reported anxiety or worry about having a kidney stone event in the future (Fig. 5D), and 13 of 16 respondents (81%) reported that pain was experienced when having a kidney stone event.

### Work productivity and activity impairment (WPAI)

**Fig. 6:**
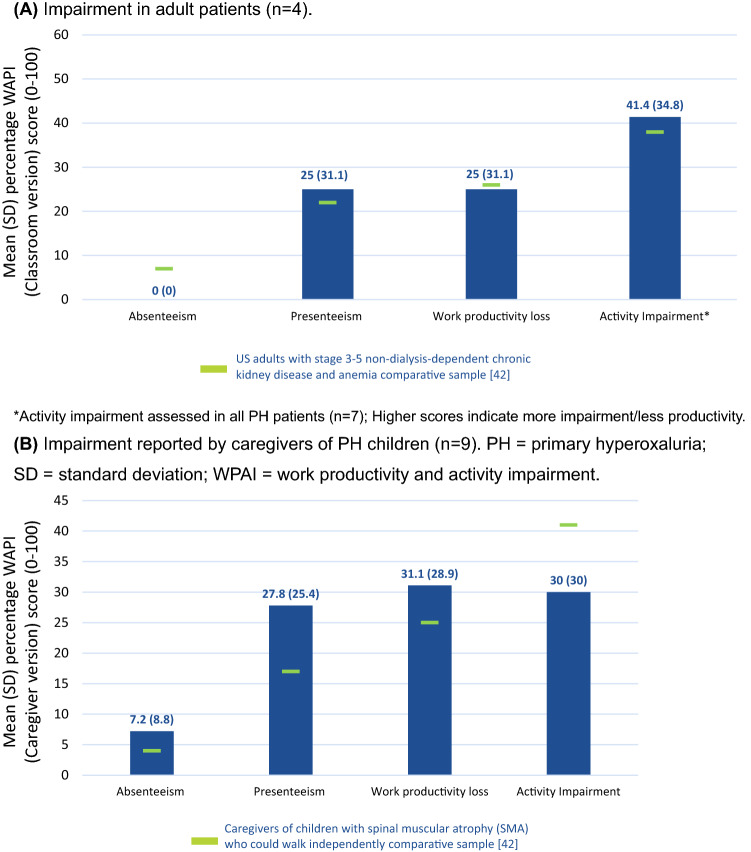
Work productivity and activity impairment for **A** adult patients with PH and **B** employed caregivers of patients with PH

Among employed patients with PH (n = 4), none reported work time lost (i.e., absenteeism) due to health, and 25% reported a moderate amount of impairment while working due to health (i.e., presenteeism) (Fig. 6). Their overall productivity loss at work was 25%. In a larger sample of evaluable respondents with PH (n = 7), activity impairment due to health was 41%.

In 9 evaluable caregivers of PH children, absenteeism, presenteeism, and overall work productivity loss due to health was 7%, 28%, and 31%, respectively. Activity impairment due to health was 30% in the 9 evaluable caregivers of PH children. Eleven of 16 survey respondents (69%) reported that PH affected their work, with respect to career choice, needing a second job, work productivity, absenteeism, and inability to work/giving up on a career.

## Discussion

This cross-sectional study of adult patients with PH and caregivers of children with PH provides a snapshot of the associated disease burden in the US. Gaining insights about the experiences of families dealing with PH is a valuable endeavor for a number of reasons. Patients and caregivers can help healthcare professionals better understand their needs, which in turn facilitates informed treatment decisions and development of clinical solutions and healthcare reimbursement plans [34–36]. Families are also in the best position to report on PH symptomatology and how their lives and livelihoods are affected by specific symptoms [37, 38]. Finally, acknowledging the gravity of the events endured by PH families enables researchers to design and execute clinical trials that are respectful of patients’ personal and medical needs, while capturing highly relevant effects of therapies in a scientifically rigorous manner [35, 36]. The supplemental appendix has a plain language summary of this manuscript to make the research more accessible to broad audiences.

First and foremost, we found that patients experience many symptoms and complications because of PH, and usually need to drink an uncomfortably high volume of fluids every day to attempt prevention of kidney stones and progressive chronic kidney disease. Hyperhydration is difficult to adhere to, time consuming, and burdensome, especially for children. As expected, all patients on dialysis (24% of the sample) found the procedure time-consuming and burdensome, while 95% of patients who had experienced kidney stone events reported on their painful, enduring nature, and the staggering number of removal procedures required. Numerous clinician visits indicated an intensive level of care. Despite patients actively managing their PH, they continue to experience negative outcomes. These findings are in full agreement with results of an international retrospective chart review of patients with PH1 under nephrologists’ care, which showed a substantial clinical burden across the spectrum of kidney impairment, including kidney stones, urinary tract infections, fatigue/weakness, and pain, for which patients require multiple urological kidney stone procedures, hospitalizations, and emergency visits [16]. Up to 1 week of school was missed every month due to PH, putting those affected further behind their peers both academically and socially.

The significant burden associated with PH may be reduced by early detection and prompt treatment. Unfortunately, our data suggest that diagnosis was a lengthy process in the period up to 2020. Over one third of respondents (38%) reported misdiagnosis, and three-quarters were diagnosed only after experiencing painful kidney stone events.

Recent progress in the treatment of patients with PH with small interfering RNA encourages early diagnosis so that optimal medical management and patient participation in relevant clinical trials can be considered [39–41]. Respondents also reported difficulty covering out-of-pocket healthcare expenses associated with PH. This finding underscores the need for data on long-term healthcare savings that could be realized from the clinical benefits provided by new PH therapies.

Comparing our HRQOL and WPAI data with other relevant patient populations provides clinical perspective. On the KDQOL-36™ questionnaire, mean PCS and MCS scores of 48 and 59, respectively, in our adult patients with PH were similar to the US general population norm and better than scores reported for a sample of adult dialysis patients in the US (PCS, 37; MCS, 49) (Fig. 5) [30]. Mean KDQOL-36™ scores for burden of kidney disease were lower in our sample than in a dialysis patient sample (38 vs. 51) but comparable regarding symptom/problem (77 vs. 78) and effects of kidney disease (65 vs. 73) [30]. Overall productivity loss at work was 25% among employed patients with PH and was 26% in a large international sample of adults with stage 3–5 non-dialysis-dependent chronic kidney disease and anemia [42]. Activity impairment due to health was 41% in respondents with PH and 38% in the chronic kidney disease population [42]. Absenteeism, presenteeism, overall work productivity loss, and activity impairment due to health was similar in caregivers of PH children and a sample of caregivers of ambulant children with spinal muscular atrophy (7% vs. 4%, 28% vs. 18%, 31% vs. 26%, and 30% vs. 41%, respectively) [43].

As seen in a separate, independent, web-based survey [14], the burden of PH goes beyond the patient to affect caregivers, as evidenced by high levels of anxiety, sleep loss, work productivity loss, and activity impairment. The prevalence of caregiver anxiety was higher in our survey than in the previous survey regarding development of stage 5 chronic kidney disease (77% vs. 24%), need for transplant (77% vs. 52%), and kidney stone event (85% vs. 36%) [14].

Due consideration is needed when interpreting the study findings beyond the standard drawbacks of collecting cross-sectional data online. The small sample size is a major limitation of this study. Inclusion of KDQOL-36™ and WPAI historical norms in other US populations were for informal context only. In our adult respondents with PH, generic measures of HRQOL using the PCS and MCS suggested comparability with a sample of adult dialysis patients in the US and the US general population norm, whereas one of the three kidney disease-specific measures of HRQOL (burden of kidney disease) suggested worse HRQOL. However, the KDQOL-36™ questionnaire, along with the WPAI, have not been specifically psychometrically validated in any PH population. Also, the KDQOL-36™ and WISQOL domain results are not norm-based. Therefore, the interpretation of domain results can lead to a misrepresentation of the stone burden. We know from using the norm-based 36-Item Short Form Survey questionnaire (SF-36) for assessing HRQOL in PH patients that, as a group, PH patients are not homogeneous and experience different HRQOL based on temporal proximity to the stone event [44]. PH type is a covariate: PH2 is associated with fewer stone events than other PH types, with an expected direct effect on HRQOL [44]. PH patients with a transplant achieve a better HRQOL than the US standard population when measured with a non-disease-specific generic instrument: most PH patients with a transplant are stone-free, with a direct effect on their HRQOL [45]. The assessment of HRQOL with a non-disease-specific instrument like the SF-36 should include additional variables like comorbidities and information about the last stone event [46]. WISQOL and SF-36 (version 2) are more appropriate for assessing HRQOL in kidney stone populations. The median out-of-pocket expenses in the past 6 months for adults ($600) and children ($2,000) appear low (range, $0–$20,000), raising the possibility that this item of the questionnaire was misinterpreted by some respondents as monthly expenses and interpreted correctly as every-6-month expenses by others. Our study did not include patients being treated with small interfering RNA. The potential effects of such therapy on the variables we measure and report in this study are not yet known.

A strength of our analysis was accounting for the PH burden via the qualitative voice response survey, with the acknowledgment that HRU, HRQOL, and WPAI impairment likely involve a combination of PH-specific and -nonspecific effects (e.g., sociodemographic issues). Repeating the survey in the era of RNA interference therapeutics is now needed, to assess whether diagnosis times and PH burden have improved.

In conclusion, this study provides new data on HRU, HRQOL, and WPAI associated with PH in patients and caregivers together with reasons for the burden. Patients experienced considerable clinical sequalae associated with PH, which appear to consume HRU and negatively affect HRQOL. PH burden also has a deleterious effect on caregivers, including anxiety and WPAI impairment. These findings highlight the need for timely PH diagnosis and improved medical management of PH, including new therapeutic strategies to better control hyperoxaluria.

### Supplementary Information

Below is the link to the electronic supplementary material.

Supplementary file1 (DOCX 17 KB)

## Data Availability

Because of the sensitive nature of the data collected for this study, the data set will not be made available to other researchers.
