# Supplementary material for: Healthcare utilization, quality of life, and work productivity associated with primary hyperoxaluria: a cross-sectional web-based US survey
Source: Urolithiasis. 2023 Apr 17;51(1):72. doi: 10.1007/s00240-023-01436-4 (PMC10110695; doi:10.1007/s00240-023-01436-4)
Supplement: Supplementary file 1 — Supplementary file1 (DOCX 17 KB) [file 240_2023_1436_MOESM1_ESM.docx]

**Supplementary Appendix**

Supplement to: David S. Goldfarb, Frank Modersitzki, John Karafilidis, Josephine Li-McLeod.

Healthcare Utilization, Quality of Life, and Work Productivity Associated with Primary Hyperoxaluria: A Cross-Sectional Web-Based US Survey.

**Plain language summary**

Primary hyperoxaluria (PH) is a group of three related, very rare, genetic diseases. For people living with PH, their bodies make too much of a natural chemical called oxalate, which can – in large enough quantities – form kidney stones. Over time, too much oxalate can also damage their kidneys and other organs. This study looked at the burden of living with PH, both as a patient and as a caregiver of children, based on responses to a web-based survey. Seven adults living with PH and 13 caregivers of 14 children living with PH responded to the survey. The answers showed that almost all (95%) people with PH had experienced at least one long-lasting, painful kidney stone. One third (33%) had visited the emergency room, and almost one third (29%) had to stay in the hospital because of PH-related issues. Nearly one quarter (24%) had to have dialysis and found the procedure burdensome. More than half (56%) found that drinking a lot of water, to help avoid kidney stones, was the most difficult thing to do. Most people (81%) answering the survey had their finances affected by PH. People also said their work productivity and activity were harmfully affected. Caregiver burden included coping with their child’s dialysis, negatively impacted sleep, and worry about future health problems related to PH. This study highlights how difficult it is to live with PH. It shows that improvements in PH care are urgently needed.

Supplemental Methods. Qualitative voice response survey.

**Patient Voice-Response Survey**

To begin, please briefly describe the process you went through to get diagnosed with PH. What type of challenges, such as time it took to get diagnosed or an initial misdiagnosis, did you experience along the way?

Next, please describe what it is like to have a kidney stone event. How does this impact your day-to-day activities? What kind of medical treatments do you need in this situation? And if you haven’t experienced a kidney stone event, how concerned are you about having one in the future?

Now, what is your current treatment regimen for PH? What challenges, if any, have you faced while trying to keep up with your current treatment? And what do you find most burdensome about it?

Next, please take a moment and think about your daily life and activities. How does PH affect aspects such as your quality of life, work productivity, relationships and finances on a day-to-day basis?

1. And finally, what challenges, if any, have you faced in managing your PH due to health insurance limitations? For example, please think about issues with access to specialists, co-pays, or aspects of your care that are not covered by your health insurance.

**Caregiver Voice-Response Survey**

To begin, please briefly describe the process your child or children went through to get diagnosed with PH. What type of challenges, such as time it took to get diagnosed or an initial misdiagnosis, did they experience along the way?

Next, please describe what it is like for your child or children to have a kidney stone event. How does this impact their day-to-day activities? What kind of medical treatments do they need in this situation? And if they have not experienced a kidney stone event, how concerned are you about them having one in the future?

Now, what is your child’s or children’s current treatment regimen for PH? What challenges, if any, have you faced while trying to keep up with their current treatment? And what do you find most burdensome about it?

Next, please take a moment to think about your family’s daily life and activities. How does PH affect aspects such as quality of life, work productivity, relationships and finances for your family?

And finally, what challenges, if any, have you faced in managing your child’s or children’s PH due to health insurance limitations? For example, please think about issues with access to specialists, co-pays, or aspects of their care that are not covered by their health insurance.
